# Detection and prevalence of antimicrobial resistance genes in *Campylobacter* spp. isolated from chickens and humans

**DOI:** 10.4102/ojvr.v84i1.1411

**Published:** 2017-05-29

**Authors:** Samantha Reddy, Oliver T. Zishiri

**Affiliations:** 1 School of Life Sciences, University of KwaZulu-Natal, South Africa

## Abstract

*Campylobacter* spp. are common pathogenic bacteria in both veterinary and human medicine. Infections caused by *Campylobacter* spp. are usually treated using antibiotics. However, the injudicious use of antibiotics has been proven to spearhead the emergence of antibiotic resistance. The purpose of this study was to detect the prevalence of antibiotic resistance genes in *Campylobacter* spp. isolated from chickens and human clinical cases in South Africa. One hundred and sixty one isolates of *Campylobacter jejuni* and *Campylobacter coli* were collected from chickens and human clinical cases and then screened for the presence of antimicrobial resistance genes. We observed a wide distribution of the *tetO* gene, which confers resistance to tetracycline. The *gyrA* genes that are responsible quinolone resistance were also detected. Finally, our study also detected the presence of the *bla*_*OXA-61*_, which is associated with ampicillin resistance. There was a higher (*p* < 0.05) prevalence of the studied antimicrobial resistance genes in chicken faeces compared with human clinical isolates. The *tetO* gene was the most prevalent gene detected, which was isolated at 64% and 68% from human and chicken isolates, respectively. The presence of *gyrA* genes was significantly (*p* < 0.05) associated with quinolone resistance. In conclusion, this study demonstrated the presence of *gyrA* (235 bp), *gyrA* (270 bp), *bla*_*OXA-61*_ and *tetO* antimicrobial resistance genes in *C. jejuni* and *C. coli* isolated from chickens and human clinical cases. This indicates that *Campylobacter* spp. have the potential of resistance to a number of antibiotic classes.

## Introduction

*Campylobacter jejuni* and *Campylobacter coli* are considered as being the most common *Campylobacter* spp. that cause gastroenteritis in humans worldwide. However, other *Campylobacter* spp. such as *Campylobacter lari* and *Campylobacter upsaliensis* have also been implicated in human gastrointestinal infections (Centers for Disease Control and Prevention [CDC] 2013; Obeng et al. 2012). There is a significant concern and paucity of information on the magnitude of antibiotic resistance patterns of *Campylobacter* spp., particularly in veterinary and human medicine (Hein et al. 2013). When infections are caused by *Campylobacter*, the symptoms are mild enteritis and are usually self-limiting that rarely requires any antimicrobial treatment. Some severe cases do, however, result in prolonged enteritis and septicaemia wherein antimicrobial treatment is essential (Chatur et al. 2014). Severe cases of *Campylobacter* infections are commonly treated with macrolides, such as erythromycin and fluoroquinolones like ciprofloxacin are used to treat enteritis. Furthermore, aminoglycosides such as gentamicin are commonly prescribed for the treatment of systemic infections (Noormohamed & Fakhr 2014). Antibiotics prescribed in veterinary medicine vary greatly throughout the world because of different regulations that are specific to countries and regions. Circumstances, where antibiotics are used for growth-promotion purposes as opposed to therapeutic purposes, are of great concern. When low levels of antibiotics are used for non-therapeutic purposes over prolonged periods of time, antimicrobial resistance emerges. Countries such as India, Indonesia, Thailand and parts of Africa have access to veterinary antibiotics without proper prescription or control measures because of the lack of properly regulated veterinary services, mainly because of scarcity of veterinary skills and training. This is of great concern and could accelerate antibiotic overuse in animals (Iovine 2013).

Molecular detection of antibiotic resistance genes has demonstrated that identical elements were found in bacteria that colonise both animals and humans. This suggests that bacteria originating from food of animal origin aid in the spread of resistant bacteria and resistance genes from animals to humans via the food chain (Moyane, Jideani & Aiyegoro 2013). Escalating numbers of *Campylobacter* isolates have developed resistance to fluoroquinolones and other antimicrobial classes such as tetracyclines, betalactams, aminoglycosides and macrolides. The acquisition of resistance mechanisms compromises treatment options against *Campylobacter* infection in both humans and animals. Although several studies have previously determined the antibiotic resistance patterns exhibited by *Campylobacter*, they have not investigated the resistance genes which are associated with drug resistant strains (Colles et al. 2016; Maćkiw et al. 2012; Ferro et al. 2015; Fonseca et al. 2014; Ghunaim et al. 2015).

Ciprofloxacin is a fluoroquinolone used for the treatment of human salmonellosis and campylobacteriosis; however, it is also used in the poultry production industry. Fluoroquinolone residues can remain in the animal body post-treatment and this aggravates the evolution fluoroquinolone resistant strains of bacteria. A number of studies have linked the therapeutic and prophylactic use of fluoroquinolone antibiotics in order to select for ciprofloxacin-resistant *Campylobacter* in poultry products that enter the food chain in an attempt to study the emergence and spread of antimicrobial resistance (Colles et al. 2016; Gallay et al. 2007; Habib et al. 2009; Marinou et al. 2012; Moyane et al. 2013; Zendehbad, Khayatzadeh & Alipour 2015).

Genetic mutations play a major role in the development of *Campylobacter* resistance. Fluoroquinolone resistance is mediated by amino acid substitutions in the quinolone resistance-determining region (QRDR) (Colles et al. 2016). The gyrase gene products are large enzymatic quaternary structures consisting of two pairs of subunits *gyrA* and *gyrB*. The *gyrA* gene, which encodes part of the *gyrA* subunit of DNA gyrase confers a high-level of resistance to ciprofloxacin because of the point mutation Thr86Ile driven by the C257T change in the *gyrA* gene. Other mutations of the *gyrA* gene region in *C. jejuni* include Thr86Ala which is responsible for high-levels of resistance to nalidixic acid and low-level resistance to ciprofloxacin (Colles et al. 2016).

Natural transformation is a major mechanism for the transfer of chromosomally encoded antibiotic resistance genes, such as for fluoroquinolone and macrolide resistance, while conjugation plays a major role in the transfer of plasmid-mediated resistance, such as for tetracyclines and aminoglycosides (Wieczorek & Osek 2013). Tetracycline resistance in *Campylobacter* spp. is conferred by the *tetO* gene, which encodes ribosomal protection proteins (RPPs). This gene is extensively present in both *C. jejuni* and *C. coli* and has been demonstrated to confer extremely high-levels of tetracycline resistance (512 mg/L) by displacing tetracycline from its primary binding site on the ribosome and thus eliminating the action of the antibiotic (Abdi-Hachesoo et al. 2014). The high prevalence of conjugative *tetO* plasmids made it possible to infer that conjugation has contributed significantly to the spread of tetracycline resistance in *Campylobacter*. Against this background, this study investigated the presence of antimicrobial resistance genes responsible for fluoroquinolone and tetracycline resistance in chicken and human clinical isolates.

## Materials and methods

### Ethical considerations

Human and animal studies were approved by the appropriate ethics committee of the University of KwaZulu-Natal and performed in accordance with the ethical standards laid down in the 1964 Declaration of Helsinki and its later amendments (REF:BE084/14).

### Origin of samples and processing procedures

A total of 100 human *Campylobacter* isolates were analysed. The samples were cryopreserved in Brucella broth (Oxoid) with 15% glycerol and were part of a collection that was received from a private laboratory in Durban, KwaZulu-Natal during 2014. One hundred faecal samples were randomly collected from commercial broiler chicken farms around the Durban metropolitan area between March and September 2016. Freshly excreted broiler faeces were sampled with sterile swabs and then directly inoculated into charcoal broth (Sigma-Aldrich, St. Louis, MO) and transported back to the laboratory for incubation at 37 °C for 48 h, under microaerophilic conditions (5% O_2_, 10% CO_2_ and 85% N_2_) created by CampyGen (Oxoid, UK) gas generating packs in an anaerobic jar.

#### Human clinical samples

From cryopreserved samples in Brucella broth (Oxoid) with 15% glycerol, cultures were revived on modified charcoal cefoperazone deoxycholate agar (mCCDA) (Blood-Free Agar) (Oxoid) containing *Campylobacter* selective supplement SR0155 (Oxoid). A sterile loop was then streaked across the area of inoculation several times to achieve isolated colonies. Thereafter, plates were incubated at 37 °C for 48 h under microaerobic conditions created by CampyGen (Oxoid) gas generating packs in an anaerobic jar. Subsequent to incubation, DNA was extracted for the purposes of species identification using the polymerase chain reaction (PCR) targeting of the *hipO* gene region (Table 1), that is the hippuricase gene specific for *C. jejuni* (Marinou et al. 2012) and the *asp* gene region (Table 1), the aspartokinase gene specific for *C. coli* (Al Amri et al. 2013).

**TABLE 1:** Target virulence genes and antimicrobial resistance genes, primer sequences, amplicon sizes and annealing temperatures.

Target gene	Primer sequence (5’ – 3’)	Product size (bp)	Annealing temperature (°C)	References
*asp*	F-GGTATGATTTCTACAAAGCGAGA R-ATAAAAGACTATCGTCGCGTG	500	53	Al Amri et al. (2007)
*hipO*	F-GAAGAGGGTTTGGGTGGT R-AGCTAGCTTCGCATAATAACTTG	735	53	Al Amri et al. (2007)
*gyrA*	F-GAAGAATTTTATATGCTATG R-TCAGTATAAC GCATCGCAGC	235	53	Chatur et al. (2014)
*gyrA*	F-ACGCAAGAGAGATGGTT R-TCAGTATAACGCATCGCAGC	270	45	Chatur et al. (2014)
*bla*_*OXA-61*_	F- AGAGTATAATACAAGCG R- TAGTGAGTTGTCAAGCC	372	54	Obeng et al. (2012)
*tetO*	F-GGCGTTTTGTTTATGTGCG R-ATGGACAACCCGACAGAAGC	559	49	Gibreel et al. (2004)

#### Chicken faecal samples

Following incubation, the faecal samples in charcoal broth (Sigma-Aldrich, St. Louis, MO) were filtered through a 0.65 **µ**m cellulose nitrate filter (Sartorius Stedim Biotech, Germany) onto mCCDA (Blood-Free Agar) (Oxoid). Approximately 500 µL of the incubated charcoal broth was evenly distributed over the filter aseptically, once the liquid had been filtered through, forceps were used to aseptically remove the filter. The culture plates were then set in an inverted position in an anaerobic jar containing an atmosphere generation system (CampyGen sachet, Oxoid) and then incubated at 37 °C for 48 h. Subsequent to incubation, DNA was isolated for the purposes of species identification using PCR targeting the *hipO* gene specific for *C. jejuni* (Marinou et al. 2012) and the *asp* gene specific for *C. coli* (Table 1) (Al Amri et al. 2013).

## DNA isolation

Template DNA for PCR was extracted via the conventional boiling method that requires the following: characteristic colonies of *Campylobacater* spp. were isolated from plates and suspended in 300 µL TE buffer, then vortexed for homogenisation of cells. The suspensions were boiled at 100 °C for 10 min, then immediately cooled on ice. After centrifugation at 14 000 × g for 5 min, the supernatants were transferred to a new tube and stored at -20 °C until use in PCR for detection of antibiotic resistance genes (Datta et al. 2003). A positive *Campylobacter* spp. control was also prepared by isolating DNA from a reference strain of *C. jejuni* ATCC 29428, which was incubated under the same conditions and subjected to the same DNA extraction methods. The Thermo Scientific Nanodrop 2000, UV-VIS Spectrophotometer (Wilmington, Delaware, USA) was used to check the concentration and quality of the isolated DNA.

## Detection of antibiotic resistance genes using polymerase chain reaction

**FIGURE 1:**
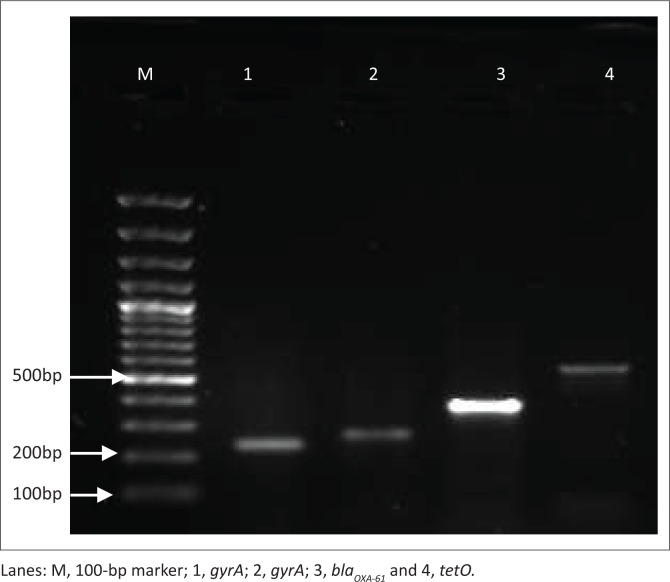
Representative gel of antibiotic resistance genes investigated from *Campylobacter* spp.

All samples were subjected to PCR analysis of the *hipO* and *asp* gene for confirmation and differentiation of *Campylobacter* spp. (Table 1). Following identification, 83% of human clinical isolates and 78% of chicken isolates were subjected to detection of the four antimicrobial resistance genes (Figure 1) as follows: *gyrA* (235 bp), *gyrA* (270 bp), *bla*_*OXA-61*_ and *tetO*. PCR primers were synthesised and sourced from Inqaba Biotechnologies (Pretoria, South Africa); forward and reverse primers specific for the antibiotic resistance genes under investigation were designed based on the gene sequence information in the GenBank database and in previously published studies (Chatur et al. 2014; Gibreel et al. 2004).

PCRs were carried out in the BIO-RAD, T100™ Thermal Cycler (Singapore) for a 25 µL reaction. The amplification conditions for *gyrA* (235 bp), *gyrA* (270 bp) and *bla*_*OXA-61*_ consisted of an initial denaturalisation at 95 °C for 5 min, 35 cycles at 95 °C for 50 s, specific Tm for each primer (Table 1) for 30 s and 72 °C for 1 min, followed by a final extension at 72 °C for 7 min. PCR conditions for the *tetO* gene, a 559 bp product, were as follows: an initial denaturalisation of 95 °C for 1 min, and then 95 °C for 1 min, 49 °C for 1 min and 72 °C for 1 min, repeated for 35 cycles. PCR products were then electrophoresed on a 1.5% agarose gel run at 60 V for 60 min, stained with ethidium bromide and then visualised using the ChemiDoc™ MP Imaging System (BIO-RAD).

## Statistical analysis of virulence genes and antibiotic resistance genes

The four antibiotic resistance genes and two virulence genes detected in *C. jejuni* and *C. coli* were analysed using IBM SPSS Statistics (Version 23). Pearson’s correlation analysis, Fisher’s exact tests, chi-square tests and logistic regression analysis were implemented to evaluate the relationship between the different PCR results obtained and the significance of the presence of virulence genes and antibiotic resistance genes detected in human clinical and chicken samples. Every model included the presence or absence of each antibiotic resistance gene and each virulence gene investigated (0 = absent; 1 = present). Associations were considered significant when *p* < 0.05.

## Results

**FIGURE 2:**
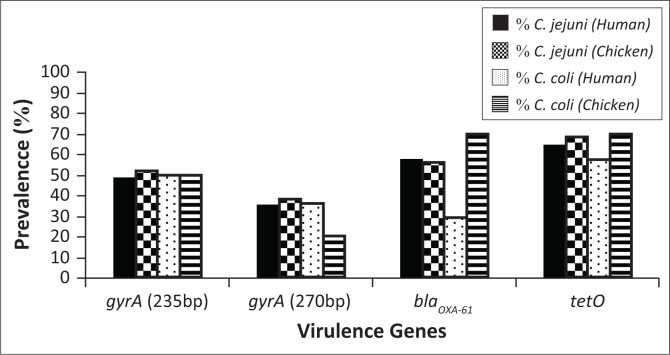
Percentage of *Campylobacter jejuni* and *Campylobacter coli*, from human clinical isolates (*n* = 83) and chicken faeces (*n* = 78) that resulted positive to each of the four antibiotic resistance genes under analysis.

Antimicrobial resistance genes isolated from *Campylobacter* spp. from human clinical samples and chicken faeces (Figure 2) demonstrated similar presence in both these hosts. The 235 bp and 270 bp *gyrA* genes were present in 49% and 36% of human clinical isolates, respectively; whereas, the *Campylobacter* from chicken faeces indicated a 51% and 36% presence of these genes, respectively, regardless of differentiation of species. The 270 bp *gyrA* gene was detected at the same incidence in both hosts. The *bla*_*OXA-61*_ gene was detected at 53% and 58% in human clinical isolates and chicken faeces, respectively. The *tetO* gene which confers tetracycline resistance was the most prevalent resistance gene detected compared with the other resistance genes under investigation. The *tetO* gene was isolated at incidences of 64% and 68% from human clinical isolates and chicken faeces, respectively. Representative PCR products of the antimicrobial resistance genes investigated in this study are depicted in Figure 1. The *gyrA* genes depicted in lanes 1 and 2 have amplicons of 235 bp and 270 bp. These confer moderate to high-levels of resistance to fluoroquinolones because of mutations occurring in these genes (Chatur et al. 2014). Lane 3 is the *bla*_*OXA-61*_ resistance gene that corresponds to a 372 bp amplicon, which confers ampicillin resistance (Obeng et al. 2012). Lane 4 is the 559 bp amplicon from the plasmid-encoded *tetO* gene, which primarily confers tetracycline resistance by displacing tetracycline from its primary binding site on the ribosome (Abdi-Hachesoo et al. 2014).

Figure 2 depicts the results which indicate that *C. jejuni* isolated from human clinical isolates, as well as chicken faecal samples, demonstrated similar presence of antimicrobial resistance genes. The *gyrA* (235 bp) and *gyrA* (270 bp) were detected at 49% and 36% in human clinical samples and 52% and 38% in chicken samples, respectively, for each gene. The resistance genes *bla*_*OXA-61*_ and *tetO* were detected at 58% and 56% in human clinical samples and 65% and 68% in chicken, respectively. Although *C. coli* was isolated at a low incidence, the antimicrobial resistance genes in this species were detected at high percentages in chicken faecal samples. Detection rates for *gyrA* (235 bp), *gyrA* (270 bp), *bla*_*OXA-61*_ and *tetO* in chicken samples were 50%, 20%, 70% and 70%, respectively. A lower incidence was observed in human clinical samples, which resulted in 50%, 36%, 29% and 57% for *gyrA* (235 bp), *gyrA* (270 bp), *bla*_*OXA-61*_ and *tetO*, respectively. There was significant association (*p* < 0.05) between all antibiotic resistance genes (*gyrA* [235 bp], *gyrA* [270 bp], *bla*_*OXA-61*_ and *tetO*) in human clinical and chicken samples investigated in this study (*p* < 0.05). This was confirmed by both the chi-square and Fisher’s exact statistical tests (Table 2).

**TABLE 2:** Results of chi-square and Fisher’s exact tests indicating *p*-values for virulence genes and antimicrobial resistance genes investigated.

Asymptotic significance (2-sided) Antibiotic resistance genes	*gyrA* (235 bp)	*gyrA* (270 bp)	*bla_OXA-61_*	*tetO*
Chi-square	0.001	0.001	0.001	0.001
Fisher’s exact test	0.001	0.001	0.001	0.001

Logistic regression analyses (Table 3) were conducted to establish the association between the presence of antimicrobial resistance genes (*gyrA, bla*_*OXA-61*_ and *tetO*), in chicken and human clinical isolates and the source of the isolates. A test of the full model against a constant only model indicated that genes *gyrA* (235 bp), *bla*_*OXA-61*_ and *tetO* were not statistically significant (*p* > 0.05) from the data obtained.

**TABLE 3:** Logistic regression analyses showing the relationship between antimicrobial resistance genes detected in *Campylobacter* spp. from human clinical isolates and chicken faeces.

Gene	B	SE	*p*	OR	95% CI
*gyrA* (235 bp)	−0.075	0.315	0.811	0.927	0.500–1.721
*gyrA* (270 bp)	0.011	0.329	0.974	1.011	0.531–1.924
*bla_OXA-61_*	−0.190	0.318	0.551	0.827	0.444–1.542
*tetO*	−0.182	0.333	0.584	0.833	0.434–1.601

B, coefficient for the constant (also called the ‘intercept’) in the null model; SE, standard error around the coefficient for the constant; *p*, probability significance value; OR, odds ratio for the independent variable X_i_ and it gives the relative amount by which the odds of the outcome increase (OR greater than 1) or decrease (OR less than 1) when the value of the independent variable is increased by one unit; 95% CI, 95% confidence interval.

Pearson correlation (Table 4) demonstrated highly significant (*p* < 0.01) positive correlations between the antibiotic resistance genes investigated in this study. High correlations existed between the ampicillin resistance gene *bla*_OXA-61_ and the tetracycline resistance gene *tetO* (64.3%), the second highest correlation was between the *tetO* gene and the *gyrA* gene for fluoroquinolone resistance (56.8%).

**TABLE 4:** Comparison of Pearson correlations coefficients for antimicrobial resistance genes.

Gene	Comparison	*gryA* (235 bp)	*gryA* (270 bp)	*bla*_OXA-61_	*tetO*
*gryA* (235 bp)	Pearson Correlation	1	0.461**	0.430**	0.568**
	Sig. (2-tailed)	-	0.000	0.000	0.000
*gyrA* (270 bp)	Pearson Correlation	0.461**	1	0.311**	0.404**
	Sig. (2-tailed)	0.000	-	0.000	0.000
*bla*_OXA-61_	Pearson Correlation	0.430**	0.311**	1	0.643**
	Sig. (2-tailed)	0.000	0.000	-	0.000
*tetO*	Pearson Correlation	0.568**	0.404**	0.643**	1
	Sig. (2-tailed)	0.000	0.000	0.000	-

** Correlation is significant at the 0.01 level (2-tailed).

## Discussion

Our results indicate that *C. jejuni* is responsible for the majority of infections (83%) in human clinical samples compared with the low incidence of *C. coli* found in 17% of cases. In chicken samples *C. jejuni* was detected at 87% compared with *C. coli* at 13%. This finding correlates with a number of studies that have demonstrated that *C. jejuni* is more prevalent than *C. coli* and, therefore, is responsible for most clinical cases related to gastroenteritis (Colles et al. 2016; Marinou et al. 2012; Moyane et al. 2013; Zendehbad et al. 2015). The increase in resistant bacteria has been associated with the injudicious use of antimicrobial agents in feed supplements used in the farming industry. This creates selective pressure, which produces microbial isolates that evolve to become resistant to these antibiotics because of the acquisition of antibiotic resistant genes. This attribute has a high likelihood of easily becoming a human health risk because of cross-transmission via the food chain (Luangtongkum et al. 2010). This study further demonstrated that higher rates of antibiotic resistance genes were detected in chicken samples when compared with the human samples. As a consequence, the injudicious use of antibiotics in chicken production accelerates the emergence of drug resistant strains of pathogens such as *Campylobacter* spp. This has a long-term effect of substantially reducing the number of antibiotics that can still be used effectively for the treatment of human and animal infections (Ferro et al. 2015). Usually it is recommended that the drug for treatment of human *Campylobacter* infections is the macrolide erythromycin, followed by ciprofloxacin of the fluoroquinolone family while the third choice would be tetracycline (Ghunaim et al. 2015).

Antibiotic resistance mechanisms for *Campylobacter* spp. differ between the drugs involved. A mutation in the *gyrA* gene acts as one of the main mechanisms of resistance for fluoroquinolones. This gene encodes part of the DNA gyrase and in the presence of a single point mutation in QRDR, codon 86 is changed from threonine to isoleucine which results in a high-level of resistance to the antibiotic ciprofloxacin (Wieczorek & Osek 2013). *Campylobacter* carrying the Thr-86-Ile change in the GyrA subunit of DNA gyrase can persist in the absence of antibiotic selection pressure. Tetracycline resistance has been shown to be typically mediated by the presence of the *tetO* gene (Abdi-Hachesoo et al. 2014).

Tetracycline has also been listed as an alternative treatment for *Campylobacter* gastroenteritis. It is used widely for therapeutic purposes in livestock and poultry, which increases the risk of tetracycline resistant *Campylobacter* spp. This study further demonstrated that high levels of the *tetO* gene which could imply that tetracycline may not be a good alternative treatment in cases of *Campylobacter* infection (Zendehbad et al. 2015). In this study, we analysed two *gyrA* genes involved in gyrase subunit A. The *gyrA* (235 bp) and *gyrA* (270 bp) were detected at 49% and 36% in human samples and 52% and 38% in chicken samples, respectively. Mutations within the *gyrA* gene lead to phenotypic expression of resistance to fluoroquinolones because of a reduced supercoiling activity of the DNA by the gyrA enzyme. This mechanism is directly linked to gene expression and therefore, the altered function of the enzyme adjusts the ‘strength’ of the resistant strains, allowing persistence of such strains even in the absence of fluoroquinolone use (Ragimbeau et al. 2014). Unlike the fluoroquinolone resistance in other organisms such as *Escherichia coli* and *Salmonella*, acquisition of high-level FQ resistance in *Campylobacter* does not necessitate stepwise accumulation of point mutations in *gyrA*. Rather, a single point mutation in the QRDR of *gyrA* gene is adequate to lead to clinically relevant levels of resistance to fluoroquinolone antimicrobials (Wimalarathna et al. 2013).

The *tetO* gene was analysed as well because the presence of this gene results in resistance by the organism to the antibiotic tetracycline (Ferro et al. 2015). Pearson correlation coefficients demonstrated significant (*p* < 0.05) correlations between the *tetO* gene and all the other antibiotic resistance genes under investigation. This study was successful in elucidating a disperse distribution of a number of antibiotic resistance genes, which play significant roles in resistance to fluoroquinolones, β-lactams and tetracycline antibiotics.

## Conclusion

This study in which the antimicrobial resistance for fluoroquinolones in *Campylobacter* spp. was screened in both human and animal samples from South Africa demonstrated that there is a correlation between the resistance genes in samples from these two species. Restricting the use of certain antibiotics in poultry production systems in South Africa may help to reduce the prevalence of antibiotic resistant *Campylobacter* strains and hence limit transmission of multi-resistant strains to the food chain. This may ultimately contribute to improving the efficacy of antibiotics used to treat human infections by controlling the emergence of resistant strains at the level of the food production industry.
